# An Anthracene-Based Hg^2+^ Fluorescent Probe with Dithioacetal: Simple Synthesis, High Selectivity and Sensitivity, and Dual-Mode Detection Capability

**DOI:** 10.3390/molecules30030561

**Published:** 2025-01-26

**Authors:** Hongli Ren, Qiang Yan

**Affiliations:** State Key Laboratory of Polymer Molecular Engineering of Polymers, Department of Macromolecular Science, Fudan University, Shanghai 200438, China; 21210440013@m.fudan.edu.cn

**Keywords:** dynamic dithioacetal reaction, anthracene, Hg^2+^, fluorescent probe

## Abstract

With the development of the chemical industry, the threat of mercury pollution to human health is increasing. Therefore, it is necessary to develop a low-cost, convenient and efficient Hg^2+^ detection method. In this study, anthracene-based Hg^2+^ fluorescent probes AN-2S and AN-4S were synthesized by a dithioacetal reaction for the rapid and efficient detection of the Hg^2+^ concentration in water. Through molecular structure design and synthesis route optimization, the complexity and cost of the probe synthesis were greatly reduced. AN-2S and AN-4S had good water solubility, rapid response abilities and anti-interference abilities, and could specifically detect Hg^2+^ using “turn-off” or “turn-on” detection modes within 1 min. The AN-4S probe showed a wide linear response range (0~40 μmol/L) and high sensitivity (4.93 × 10^−8^ mol/L) to Hg^2+^ in 99% aqueous solutions, over a pH range of 5~13. The reaction mechanism between the probe and Hg^2+^ was determined using 1H NMR and FT-IR spectra and Job’s curves. It was proven that the AN-2S and AN-4S probes react with Hg^2+^ in a molar ratio of 1:1 or 1:2. The dual-detection mode enabled the probes to be used not only for the accurate quantitative detection of Hg^2+^ under a fluorescence spectrometer, but also for rapid qualitative analysis using a UV flashlight as a test strip, showing a broad practical application potential.

## 1. Introduction

With the advancement of industrialization, particularly in chemical manufacturing, fossil fuel burning and mining, the threat of mercury pollution to human health has increased significantly. Mercury has a high affinity for various active groups such as sulfhydryl, amino and carbonyl groups in organisms, and is one of the most toxic heavy metal pollutants [1,2,3,4]. More seriously, as a heavy metal element that cannot be biodegraded, mercury will gradually accumulate along the food chain [5,6,7,8,9], causing serious harm to humans at the top of the food chain [10,11,12,13,14]. In order to detect the mercury content in the environment, atomic absorption spectrometry [15], inductively coupled plasma mass spectrometry [16,17], atomic fluorescence spectrometry [18], chemical sensor methods [19] and other detection technologies have been developed. However, these methods generally have disadvantages such as complex pretreatment procedures, high reagent costs and expensive fixed instruments, which make it difficult to detect Hg^2+^ quickly, efficiently and cheaply.

In recent years, fluorescent probes have been regarded as an important research direction in the field of metal ion detection because of their high selectivity, real-time analysis capability and simple operation. A variety of probes targeting mercury ions have been developed. Based on their working mechanisms, these probes can be classified into three categories: “turn-off” probes, “turn-on” probes and ratiometric probes. “Turn-off” probes refer to probes that exhibit a significant reduction in fluorescence intensity or complete quenching in the presence of mercury ions [20,21]. These probes show a pronounced fluorescence quenching effect and a rapid reaction rate. In contrast, “turn-on” probes refer to probes that display a significant enhancement in fluorescence intensity or new emission peaks in the presence of mercury ions [22,23,24,25]. These probes are characterized by a high signal-to-noise ratio and high sensitivity. Ratiometric probes detect mercury ions by monitoring the ratio change of the intensity of multiple emission peaks [26,27]. This type of probe has self-calibration characteristics, which can effectively eliminate background interference and improve detection accuracy.

However, the widespread application of fluorescent probes still faces some challenges. First of all, the molecular design and synthesis route of most probes are complicated, which not only increases the difficulty of synthesis, but also increases the cost. Secondly, most existing fluorescent probes are limited to a single detection mode, with relatively fixed excitation and emission wavelengths. In complex biological or environmental systems, the detection sensitivity can be significantly impacted by the presence of other fluorescent substances with similar emission wavelengths. Therefore, the development of fluorescent probes with a variety of detection modes and flexibility in the choice of a working wavelength is of great significance for avoiding background fluorescence interference and realizing the universal detection of mercury ions. This not only helps to improve the accuracy and reliability of detection, but also provides a possibility for the application of fluorescent probes in a wider range of fields.

Dithioacetal bonds are a kind of dynamic covalent bond which have been given more attention in recent years. Because of its low bond energy, dithioacetal bonds are more likely to have specific devulcanization reactions with Hg^2+^, making them suitable as the response group of Hg^2+^ fluorescent probes. Furlan et al. proposed a dynamic dithioacetal reaction catalyzed by a Lewis acid in 2016 [28,29,30,31]. This reaction has the advantages of mild conditions, a high reaction rate and a high functional group tolerance, which reduced the difficulty of synthesis of dithioacetal-type probes. In this study, the dynamic dithioacetal reaction was used as a tool to prepare the AN-2S and AN-4S probes with multiple dithioacetal sites in one step. After reacting with Hg^2+^, stable S-Hg-S complexes were formed, and the conjugated and electron-absorbing aldehyde groups were exposed. This reaction is conducive to the formation of the intramolecular charge transfer mechanism (ICT) in the fluorophore, which affects the fluorescence emission behavior of the molecules. The fluorescence emission characteristics of the AN-4S probe and its reaction product AN-DC exhibit significant differences. By individually monitoring the changes in fluorescence intensity (enhancement or quenching) of these two molecules, a linear response to the mercury ion concentration can be achieved, endowing the system with the unique advantage of dual detection modes. After the modification of multiple carboxyl groups, the water solubility of the probe was significantly improved, and its solvent environment was more consistent with a natural water environment. The fitting curve and linear response range of the probe were measured using a fluorescence titration experiment, and the response ability of the probe was verified under different pH and interfering-ion conditions. The mechanism of the probe’s reaction with Hg^2+^ was explained using NMR FT-IR spectroscopy and density functional theory (DFT) simulations. In order to explore the practical application of the probe, this study validated the ability of the probe to achieve qualitative or quantitative detection of Hg^2+^ under natural conditions. In comparison to similar studies, the probe showed several advantages, including a straightforward synthesis, excellent water solubility, a rapid response, strong selectivity, high environmental tolerance, exceptional sensitivity, and a broad linear range, highlighting its significant potential for practical applications.

## 2. Results and Discussion

### 2.1. Synthesis of Water-Soluble AN-2S and AN-4S Probes

**Scheme 1:**
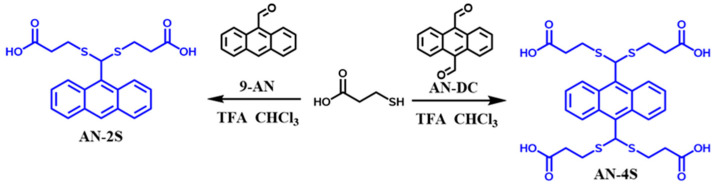
Synthesis of water-soluble AN-2S and AN-4S probes.

The AN-2S and AN-4S probes were synthesized from commercially available raw materials using the dynamic dithioacetal reaction as a tool. As shown in Scheme 1, mercaptopropionic acid and the fluorescent molecule 9-AN or AN-DC with aldehyde groups were dissolved in CHCl_3_, and the dithioacetal reaction occurred under the catalysis of TFA. The water-soluble AN-2S and AN-4S probes were synthesized within 30 min. This synthesis route required only one chemical reaction and no strict water or oxygen removal steps, which greatly reduced the difficulty and cost of synthesis of the probes (detailed synthesis steps are shown in Section 3).

### 2.2. Solvent and Detection Mode Selection

**Figure 1:**
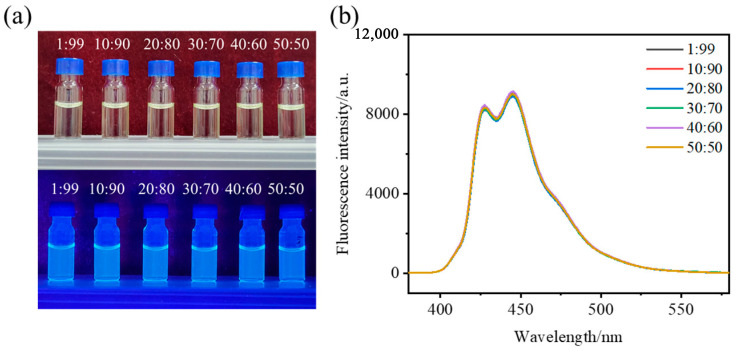
(**a**) Fluorescence image and (**b**) fluorescence spectra of AN-4S probe (100 μmol/L) in different solvents (*λ*_ex_ = 360 nm, *V*(DMF): *V*(PBS) = 1:99, 10:90, 20:80, 30:70, 40:60, and 50:50).

Due to the large cyclic conjugated structure of fluorophores, some probes have poor water solubility and need to be dissolved in a large proportion of organic solvents [32,33]. Fluctuations in the solvent ratio can affect the degree of aggregation of these probes, which can lead to instability in their fluorescence properties and reduce the accuracy of their detection. In order to overcome this problem, several carboxyl groups were introduced into the side group of the probes, which greatly enhanced the water solubility of the probes. According to fluorescence experiment, the probes have good water solubility. AN-2S can be stably dissolved in a solvent with a maximum water content of 90%, and has the strongest fluorescence emission capacity in this solvent system (Figure S11). Compared with AN-2S, AN-4S was expected to have better water solubility and a higher detection sensitivity due to more dithioacetal sites and water-soluble carboxyl groups. AN-4S can be dissolved in a solvent with a 99% water content, and showed uniform dispersion and a stable fluorescence emission ability in various solvent systems (Figure 1). Therefore, the mixed solvent shown in Table 1 was selected as the solvent for the two probes.

**Figure 2:**
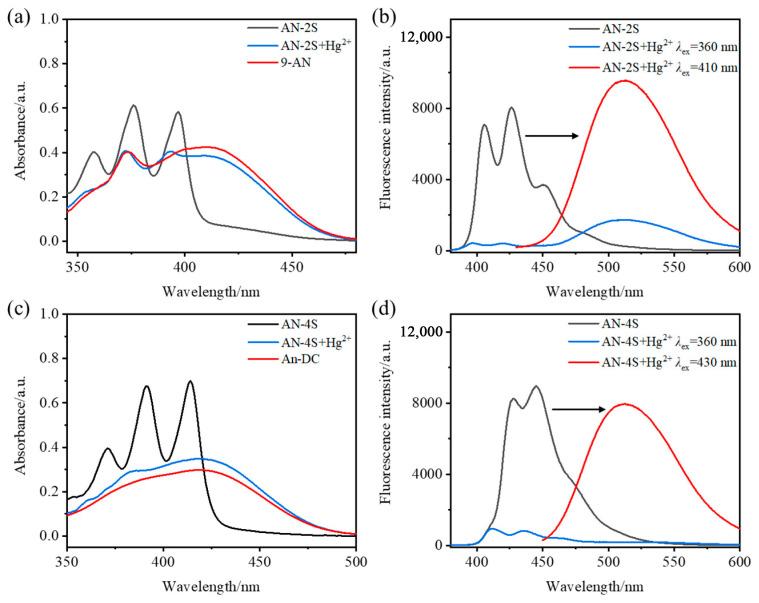
(**a**) UV–vis absorption and (**b**) fluorescence spectra of AN-2S (100 μmol/L) before and after reaction with Hg^2+^ (100 μmol/L); (**c**) UV–vis absorption and (**d**) fluorescence spectra of AN-4S (100 μmol/L) before and after reaction with Hg^2+^ (200 μmol/L).

The operating wavelength and detection mode of the probe were determined using the UV–vis absorption and fluorescence emission spectra before and after the reaction. As shown in Figure 2, AN-2S and AN-4S had obvious UV absorption peaks in the range of 360~430 nm, which corresponded to fluorescence emission at 400~475 nm. Upon rapid reaction with excess Hg^2+^, the probe exhibited a significant decrease in its original absorption peak. Concurrently, a new absorption peak attributed to anthracene emerged within the 410~430 nm range, which correlated with the fluorescence emission peak observed between 510 and 530 nm. The spectra before and after the reaction showed obvious redshift, and the stokes shift was between 80 and 100 nm, which helped to improve the signal-to-noise ratio and ensure the accuracy and reliability of experimental results. As shown in Table 1, the probes had “turn-off” and “turn-on” detection modes according to the change in the fluorescence emission before and after the reaction, which can detect Hg^2+^ at different excitation wavelengths (*λ*_ex_).

### 2.3. Fluorescence Titration Experiment

**Figure 3:**
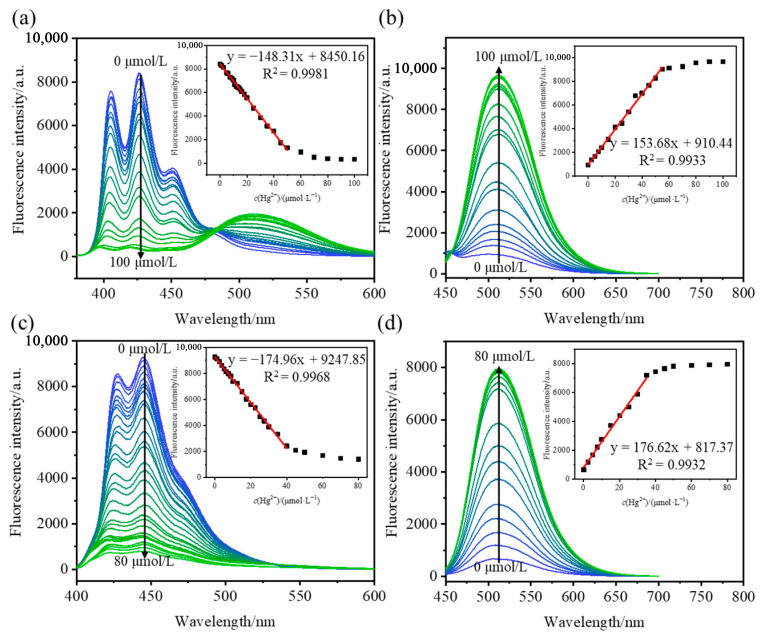
(**a**) “Turn-off” fluorescence spectra and fitting curve of AN-2S with Hg^2+^ (*λ*_ex_ = 360 nm, *c*(AN-2S) = 100 μmol/L, *c*(Hg^2+^) = 0~100 μmol/L); (**b**) “turn-on” fluorescence spectra and fitting curve of AN-2S with Hg^2+^ (*λ*_ex_ = 410 nm, *c*(AN-2S) = 100 μmol/L, *c*(Hg^2+^) = 0~100 μmol/L); (**c**) “turn-off” fluorescence spectra and fitting curve of AN-4S with Hg^2+^ (*λ*_ex_ = 360 nm, *c*(AN-4S) = 100 μmol/L, *c*(Hg^2+^) = 0~80 μmol/L); (**d**) “turn-on” fluorescence spectra and fitting curve of AN-4S with Hg^2+^ (*λ*_ex_ = 430 nm, *c*(AN-4S) = 100 μmol/L, *c*(Hg^2+^) = 0~80 μmol/L).

Fluorescence titration experiments were conducted to draw the fitting curves of AN-2S and AN-4S in different detection modes. As shown in Figure 3, both probes (100 μmol/L) exhibited a linear response to varying Hg^2+^ concentrations over a wide range in both the “turn-off” and “turn-on” detection modes. The working curves under the different detection modes can be obtained by fitting the Hg^2+^ concentration as the abscissa and the fluorescence intensity as the ordinate. According to the standard detection of limit (LOD) formula [34]

LOD = 3σ/S(1)

the LOD of the probe under different detection modes can be calculated, where σ is thestandard deviation of 20 measurements of the blank response, and S is the slope of the fitting curve (Table 2). According to the calculation results, the two probes had lower detection limits in the “turn-on” mode, and the lowest LOD reached 4.9 × 10^−8^ mol/L. The detection limit in the “turn-off” mode was slightly higher, around 2.5 × 10^−7^ mol/L. According to China’s Sanitary Standards for Drinking Water, the concentration of Hg^2+^ in surface water should not exceed 0.05 mg/L (2.5 × 10^−7^ mol/L) [35]. Therefore, the LODs of the two probes in different detection modes were lower than the maximum allowable concentration of Hg^2+^ in surface water in China, which could be used to detect Hg^2+^ in water.

The difference in the LOD of the probes in the two detection modes is mainly caused by instrument error. For AN-4S, the initial fluorescence intensity in the “turn-off” mode was high, and the standard deviation caused by the numerical fluctuation was large (σ = 16.83), which greatly affected the detection accuracy. However, the low initial fluorescence intensity in the “turn-on” mode resulted in a smaller standard deviation (σ = 2.92), so the accuracy was higher. The two detection modes have their own application scenarios. We can use a common 365 nm UV lamp for the rapid qualitative detection of Hg^2+^, and we can also use a fluorescence spectrometer for the accurate characterization of Hg^2+^ concentrations.

### 2.4. Selectivity and Anti-Interference Ability

**Figure 4:**
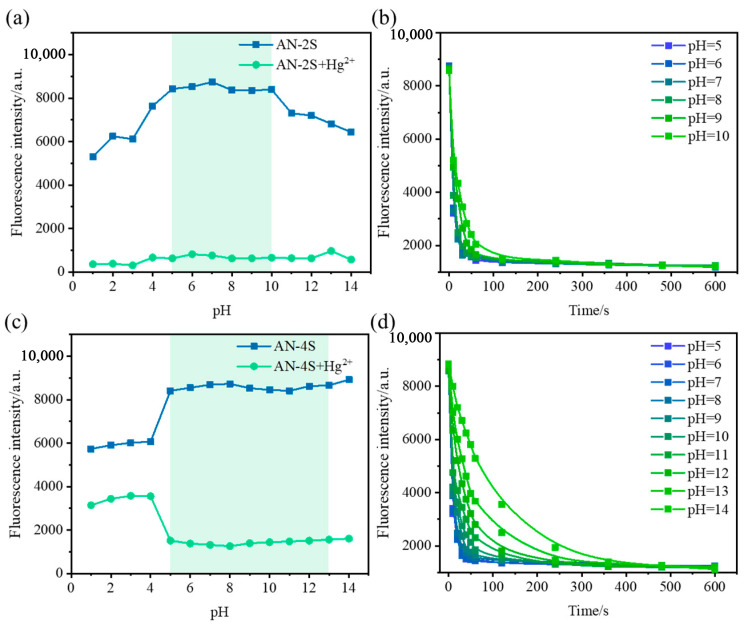
Fluorescence response of (**a**) AN-2S (*λ*_ex_ = 360 nm, *c*(AN-2S) = 100 μmol/L, *c*(Hg^2+^) = 100 μmol/L) and (**c**) AN-4S (*λ*_ex_ = 360 nm, *c*(AN-4S) = 100 μmol/L, *c*(Hg^2+^) = 200 μmol/L) under different pH conditions; impact of time on the fluorescence intensity of (**b**) AN-2S (*λ*_ex_ = 360 nm, *c*(AN-2S) = 100 μmol/L, *c*(Hg^2+^) = 100 μmol/L) and (**d**) AN-4S (*λ*_ex_ = 360 nm, *c*(AN-4S) = 100 μmol/L, *c*(Hg^2+^) = 200 μmol/L) for Hg^2+^ under different pH conditions.

In real-world applications, the sample compositions are often more complex than under laboratory conditions, with potential variations in acidity, alkalinity or the presence of various metal ions. Therefore, it is essential that the probe exhibits strong selectivity and anti-interference capabilities. When pH = 5~10, the fluorescence intensity of AN-2S was high (Figure 4a), and the fluorescence was quenched within 1 min after the addition of Hg^2+^ (Figure 4b). Compared with AN-2S, the AN-4S probe had a wider pH application range, and its fluorescence emission spectrum at different pH is shown in Figure 4c. When pH = 5~14, the fluorescence intensity of AN-4S was high, and the fluorescence was rapidly quenched after the addition of Hg^2+^.When the pH value was less than 4, the fluorescence intensity of AN-4S decreased significantly and was less affected by the addition of excess Hg^2+^. This may due to the protonation of the probe under acidic conditions, which led to a weakening of the probe’s ability to respond to Hg^2+^. When pH = 5~11, the fluorescence intensity of AN-4S rapidly decreased to the minimum within 1 min after the addition of Hg^2+^ (Figure 4d), indicating that it reacted almost completely with Hg^2+^ in the system within 1 min. When pH = 12~13, the probe completely reacted with mercury within 2~4 min, which can barely meet the requirements for rapid detection. When pH = 14, the reaction was basically completed in 8~10 min, which did not meet the needs requirements for rapid detection. The slower reaction rate of AN-4S in the environment with a higher pH may be due to the presence of more carboxylic acid groups in the structure. In a more alkaline environment, the ionized carboxylate ions will be affected by electrostatic repulsion when they react with other molecules, so the reaction rate will be reduced. Therefore, considering the influence of pH on the fluorescence emission intensity and response speed, the pH application range of AN-4S was determined to be 5~13.

Moreover, we tested the fluorescence properties of the probes at different temperatures (Figure S12), after prolonged exposure to natural conditions (Figure S19) and after ultraviolet illumination (Figure S20), and demonstrated that the probe maintained good stability under various conditions.

**Figure 5:**
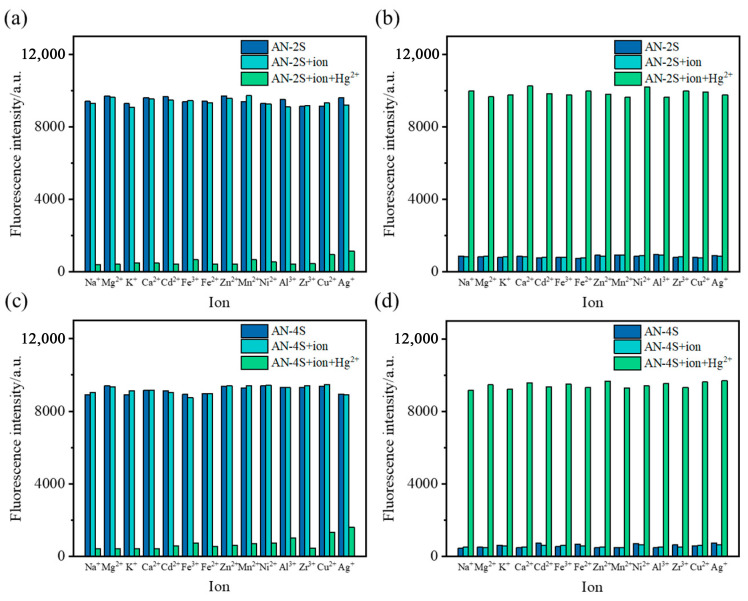
Influence of ions on AN-2S (*c*(AN-2S) = 100 μmol/L, *c*(ion) = *c*(Hg^2+^) = 100 μmol/L) when (**a**) *λ*_ex_ = 360 nm and (**b**) *λ*_ex_ = 410 nm; influence of ions on AN-4S (*c*(AN-4S) = 100 μmol/L, *c*(ion) = *c*(Hg^2+^) = 200 μmol/L) when (**c**) *λ*_ex_ = 360 nm and (**d**) *λ*_ex_ = 430 nm.

The AN-2S and AN-4S probes had good selectivity. As shown in Figure 5, the fluorescence intensity of the probes (*λ*_ex_ = 360 nm and 410/430 nm) did not change significantly after the addition of different metal ions. The fluorescence was quenched within 5 min after the addition of Hg^2+^, which proved that the probes specifically recognized Hg^2+^. In addition, the probes also showed good selectivity for Hg^2+^ even with interference from various anions (Figures S13 and S14).

### 2.5. Response Mechanism

**Figure 6:**
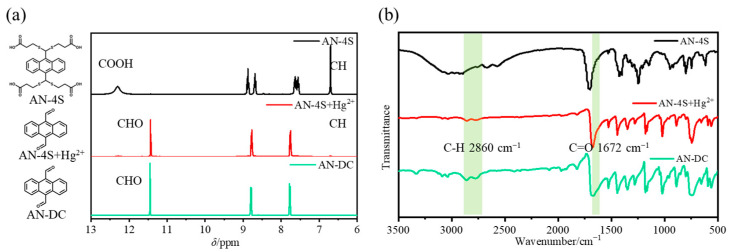
(**a**) ^1^H NMR and (**b**) FT-IR spectra of AN-4S, AN-4S+Hg^2+^ and AN-DC.

The response mechanism of the probe was studied by 1H NMR and FT-IR spectroscopy. In Figure 6a, the AN-4S probe showed a distinct single peak from the H on dithioacetal at 6.7 ppm. After the reaction with excess Hg^2+^, the dithioacetal peak in the hydrogen spectrum of the product disappeared, and a sharp peak from the H on the aldehyde group appeared at 11.5 ppm. The 1H NMR spectra of the product were consistent with that of AN-DC (Figure 6a). It can also be observed from the FT-IR spectrograms in Figure 6b that the stretching vibration peaks of the O-H bond (3000 cm^−1^) and C=O bond (1710 cm^−1^) on the carboxyl group of the probe disappeared after sufficient reaction with Hg^2+^. The stretching vibration peaks of the C=O bond (1672 cm^−1^) and the C-H bond (2860 cm^−1^) on the aldehyde group appeared in the spectra. The FT-IR spectra of the product were also consistent with that of AN-DC (Figure 6b). The 1H NMR and FT-IR characterization proved that the dithioacetal group on the probe had completely desulfurized with Hg^2+^, and the product was AN-DC. Similarly, the representation in Figure S15 also proved that AN-2S generated 9-AN after a complete desulfurization reaction.

**Figure 7:**
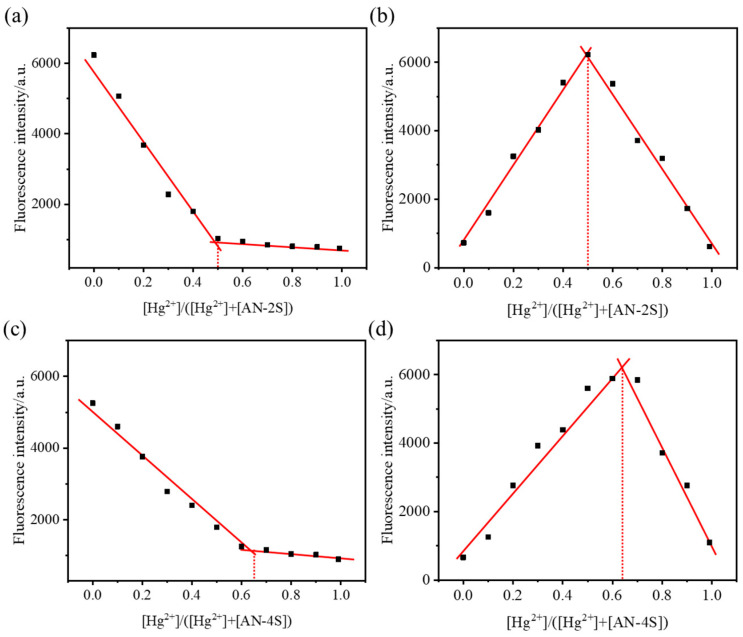
Job’s blot of AN-2S (*c*(AN-2S) + *c*(Hg^2+^) = 50 μmol/L) at excitation wavelengths of (**a**) 360 nm and (**b**) 410 nm; Job’s blot of AN-4S (*c*(AN-4S) + *c*(Hg^2+^) = 50 μmol/L) at excitation wavelengths of (**c**) 360 nm and (**d**) 430 nm.

**Scheme 2:**
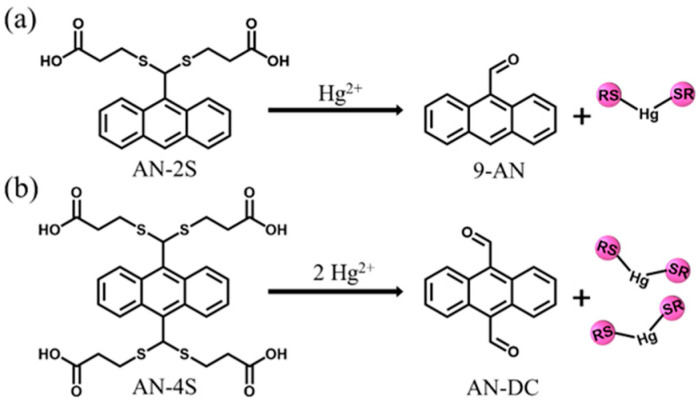
Mechanism of the response of (**a**) AN-2Sand (**b**) AN-4S to Hg^2+^.

The stoichiometric ratios of AN-2S and AN-4S after Hg^2^ reactions were determined using a Job’s blot test. The total concentration of the probe and Hg^2+^ was fixed at 50 μmol/L while the ratio of the two was changed, and the fluorescence intensity in the “turn-on” detection mode was recorded to obtain the Job’s blot of the probes. As shown in Figure 7a,b, the fluorescence intensity of AN-2S reached its peak when [Hg^2+^]/([Hg^2+^] + [AN-2S]) = 0.5, so the molar ratio of AN-2S to Hg^2+^ was 1:1, which proved that the stoichiometric ratio of AN-2S to Hg^2+^ was 1:1. For AN-4S, the fluorescence intensity of the system reached a peak when [Hg^2+^]/([Hg^2+^] + [AN-4S]) = 0.67, and the stoichiometric ratio of the probe to Hg^2+^ reaction was 1:2 (Figure 7c,d). The response mechanisms of the two probes are shown in Scheme 2. The dithioacetal site in the probe reacted with Hg^2+^ in a molar ratio of 1:1. AN-4S had twice as many stimulus-responding groups as AN-2S, so it should also theoretically be more sensitive.

**Figure 8:**
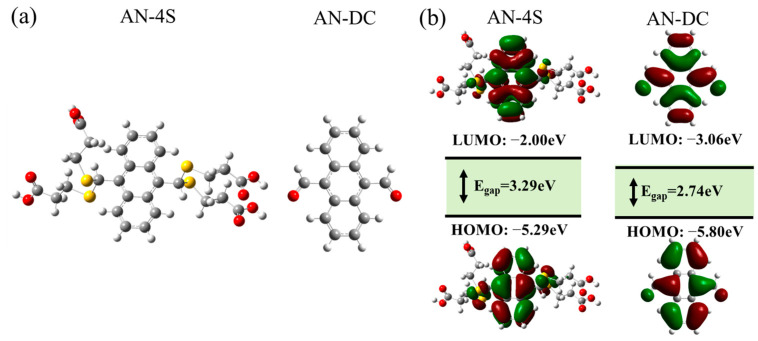
(**a**) Optimized molecular configurations for AN-4S and AN-DC probes; (**b**) HOMO–LUMO energy gap between AN-4S and AN-DC probes.

The HOMO–LUMO energy range of the probe before and after the reaction was calculated using DFT molecular simulations. The calculation results showed that the energy difference between the HOMO and LUMO of AN-DC after the desulfurization reaction was smaller than that of the AN-4S probe (Figure 8). This may be due to the strong conjugation effect of the aldehyde groups exposed after the molecular desulfurization reaction, which led to a reduction in the energy required for intergap transitions, which then caused a redshift of the absorption spectrum. This was in accordance with the redshift phenomenon of the UV absorption and fluorescence emission spectra in the experiment. A consistent conclusion was also obtained for the calculation results for AN-2S and 9-AN (Figure S18).

### 2.6. Practical Application

**Figure 9:**
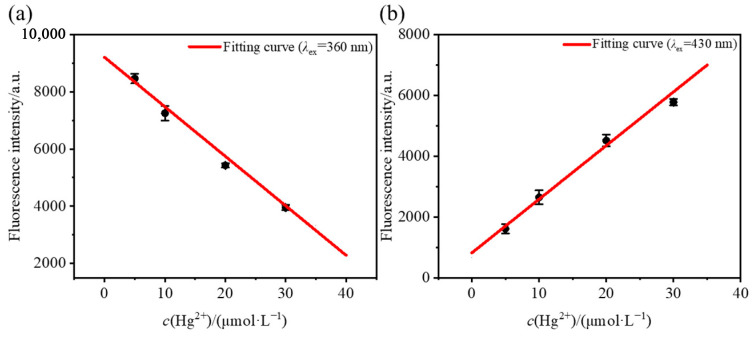
Response diagram of AN-4S (100 μmol/L) to Hg^2+^ in simulated natural water samples when (**a**) *λ*_ex_ = 360 nm and (**b**) *λ*_ex_ = 430 nm.

The Hg^2+^ detection ability of the probes was explored in simulated natural water samples. Based on the fitting curve for the AN-4S probe, the Hg^2+^ concentration in the simulated water samples was characterized under two detection modes (each group was measured three times and averaged). The results are shown in Figure 9 and Table 3 (the results for AN-2S are shown in Figure S16 and Table S1). The characterization results showed that AN-4S had a relatively high recovery rate for Hg^2+^ concentration in each group of samples under the double-detection conditions. This indicates that the high sensitivity, high selectivity and wide pH adaptation range of the probe enabled it to overcome various interfering factors in the natural environment and detect the Hg^2+^ concentration in natural water samples quickly and with high precision.

**Figure 10:**
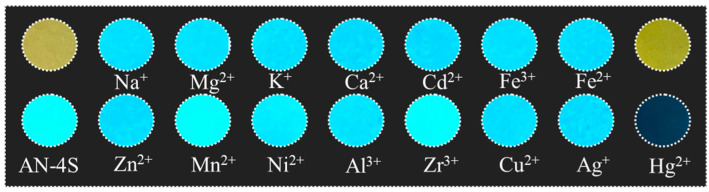
Images of the AN-4S test strip under sunlight and 360 nm UV light before and after immersed in water samples.

The dual-detection mode of the probe also makes its application scenarios more diverse. Without a fluorescence spectrometer, the probe can be prepared as a portable test strip for the rapid qualitative detection of Hg^2+^ in water samples using a common 365 nm UV lamp. The fluorescence images in Figure 10 and Figure S17 proved that the test strip had good anti-interference ability to various metal ions, and the presence of metal ions did not affect the response to Hg^2+^. After immersion in water samples without additional Hg^2+^, the strips maintained their blue fluorescence under 365 nm UV light. However, when immersed in natural water samples with Hg^2+^ added, the fluorescence of the probe under the UV lamp disappeared.

### 2.7. Comparison with Other Probes

Compared with similar probes that have been proposed [32,33,34,36,37,38], the probes designed in this study have excellent comprehensive performance (Table 4). Compared with the complex structure and synthetic steps of similar probes, AN-2S and AN-4S have simple structures and can be synthesized by a one-step chemical reaction, which greatly improves the operability of the probes in a wide range of practical applications. The probes have the advantages of high sensitivity, good water solubility and a wide response range, which can meet the demand for water sample detection under natural conditions.

## 3. Materials and Methods

### 3.1. Reagents and Instruments

9-Anthraaldehyde (9-AN, purity: 99.0%), 9, 10-anthradialdehyde (AN-DC, purity 99.0%), 4-mercaptopropionic acid (purity: 99.0%) and trifluoroacetic acid (TFA, purity 99.0%) were purchased from Bide Pharmatech Ltd. (Shanghai, China). Trichloromethane (purity: 99.0%), anhydrous magnesium sulfate (purity: 98.0%), N, n-dimethylformamide (DMF, purity: 99.0%), sodium hydroxide (NaOH, purity: 96.0%), hydrochloric acid (HCl, purity: 36.0–38.0%) and phosphate buffer (PBS, purity: 98.0%, pH = 7.4) were purchased from Sinopharm Chemical Reagent Co., Ltd. (Beijing, China). The instruments used were an AVANCE III HD-400 Nuclear Magnetic Resonance (NMR) Spectrometer (Bruker, Billerica, MA, USA); AB SCIEX 5800 Matrix-Assisted Laser Desorption Time-of-Flight Mass (MALDI-TOF) Spectrometer (SCIEX, Framingham, MA, USA); Cary 6000i Ultraviolet–Visible (UV–vis) Spectrophotometer (Agilent Corporation, Santa Clara, CA, USA); RF-5301PC Fluorescence (FL) Spectrometer (Shimadzu, Kyoto, Japan); and Nicolet 670 Fourier Transform Infrared (FT-IR) Spectrometer (Thermofisher, Waltham, MA, USA).

### 3.2. One-Step Synthesis of Fluorescent AN-2S Probe

9-AN (2.06 g, 10.0 mmol), mercaptopropionic acid (2.12 g, 20.0 mmol) and chloroform (30 mL) were added to a dry 50 mL flask and stirred. After the system became transparent, TFA (3 mL) was slowly added to the bottle. The system was heated to 50 °C and reacted for 30 min under stirring. It was cooled to room temperature and filtered to obtain a yellow-green solid. This solid was dissolved in chloroform (20 mL) and extracted three times with an aqueous solution of sodium hydroxide (1 mg/mL, 10 mL) to merge the aqueous phase. The aqueous phase was acidified by adding an aqueous solution of hydrogen chloride (1 mg/mL, 30 mL) and extracted three times through chloroform (20 mL) to merge the organic phase. After adding anhydrous magnesium sulfate (20 g) and drying for 1 h, the solvent was filtered and drained, and 3.24 g of dark yellow powder product was obtained after vacuum drying, with a yield of 81.0%. ^1^H NMR (400 MHz, DMSO-*d*_6_, 298 K): δ = 12.29 (s, 2H), 8.84 (m, 1H), 8.61 (m, 2H), 8.11 (m, 2H), 7.63 (m, 1H), 7.55 (m, 3H), 6.70 (s, 1H), 2.91 (m, 4H), 2.55 (m, 4H). ^13^C NMR (100 MHz, DMSO-*d*_6_, 298 K): δ = 170.7, 131.0, 130.2, 129.1, 127.5, 126.7, 125.7, 38.0, 34.9, 23.2; MALDI-TOF (C_21_H_20_O_4_S_2_ theoretical value), *m*/*z*: 400.27(400.38), 423.19[M + Na^+^]. The ^1^H NMR, ^13^C NMR and MALDI-TOF spectra are shown in Figures S1, S2 and S9.

### 3.3. One-Step Synthesis of Fluorescent AN-4S Probe

AN-DC (1.17 g, 5.0 mmol), mercaptopropionic acid (2.12 g, 20.0 mmol) and chloroform (24 mL) were added to a dry 50 mL flask and stirred. After the system became transparent, TFA (3 mL) was slowly added to the bottle. The system was heated to 50 °C and reacted for 30 min under stirring. It was cooled to room temperature and filtered to obtain a yellow-green solid. This solid was dissolved in chloroform (20 mL) and extracted three times with an aqueous solution of sodium hydroxide (1 mg/mL, 10 mL) to merge the aqueous phase. The aqueous phase was acidified by adding an aqueous solution of hydrogen chloride (1 mg/mL, 30 mL) and extracted three times through chloroform (20 mL) to merge the organic phase. After drying with anhydrous magnesium sulfate (20 g) for 1 h, the solvent was filtered and drained, and the dark yellow powder product (2.68 g) was obtained after vacuum drying, with a yield of 86.1%. ^1^H NMR (400 MHz, DMSO-*d*_6_, 298 K): δ = 12.30 (s, 4H), 8.87 (m, 4H), 7.64 (m, 4H), 6.70 (s, 2H), 2.93 (m, 8H), 2.56 (m, 8H); ^13^C NMR (100 MHz, DMSO-*d*_6_, 298 K): 170.3, 134.3, 129.7, 128.2, 124.6, 38.0, 35.0, 23.3; MALDI-TOF(C_28_H_30_O_8_S_4_ theoretical value), *m*/*z*: 622.23(622.08), 645.58[M + Na^+^]. The ^1^H NMR, ^13^C NMR and MALDI-TOF spectra were shown in Figures S5, S6 and S10.

### 3.4. Preparation of Test Samples

The probe was dissolved in DMF to obtain a probe mother solution (1.0 × 10^−2^ mol/L). The probe mother liquor was added into the mixed solvent of DMF and water at different volume ratios and diluted to 100 μmol/L, and after adding a certain amount of Hg^2+^, the test solution for the fluorescence intensity test was obtained after 5 min. A certain amount of a HCl or NaOH solution was added to PBS to adjust the pH value, and DMF was mixed with water samples in the same proportion as the added probes (100 μmol/L), and a certain amount of Hg^2+^ was added and reacted for 5 min to obtain the samples of probes under different pH conditions. In the selective experiment, different interfering ions were mixed with the probe for 5 min and then characterized.

### 3.5. Preparation of Simulated Natural Water Samples

Lake water from Xiyuan was used as the water sample to be tested, and the insoluble matter was removed by sedimentation and filtration. DMF was mixed with the treated lake water in a certain proportion [for AN-2S: *V*(DMF):*V*(H_2_O) = 1:9; for AN-4S: *V*(DMF):*V*(H_2_O) = 1:99], and a certain amount of Hg^2+^ was added to obtain simulated natural water samples with different concentrations of Hg^2+^. The recovery rate of the probes to Hg^2+^ in natural water samples could be determined by adding the probes (100 μmol/L) to the simulated water sample and measuring their fluorescence emission spectrum after mixing for 1 min.

### 3.6. Preparation of Portable Test Strip

The filter paper splines were immersed in the probe mother solution for 10 min, removed, and dried, and thus the portable Hg^2+^ test strip was prepared.

## 4. Conclusions

In this study, anthracene was used as the fluorophore and a dynamic dithioacetal reaction was used as the synthesis method to prepare the dithioacetal-type fluorescent AN-2S and AN-4S probes through a one-step synthesis process. The probes had good water solubility and are suitable for the water environments in nature. The probes had two detection modes of “turn-off” and “turn-on”, and their fluorescence intensities showed a good linear relationship with Hg^2+^ concentration. The LOD of the probes reached 4.8 × 10^−8^ mol/L, and the linear response range was 0~40 μmol/L. They are at the forefront of this type of probe. The probes had a good anti-interference ability, can be used to quantitatively detect Hg^2+^ in water environments within 1 min, and can be prepared into test strips for qualitative analyses. The reaction principle of the probe with Hg^2+^ and the change in its optical properties were explained using NMR, FT-IR, and DFT analyses. The probe has the advantages of a simple structure, easy synthesis, good environmental adaptability, rapid response, high sensitivity, a wide detection range, a strong anti-interference ability, and excellent comprehensive performance. However, the high hydrophilicity of the AN-4S probe may result in its inability to enter the cell membrane, thus limiting its biological application. Improvements need to be made in subsequent studies so that the probe can be applied in more scenarios.

## Figures and Tables

**Table 1:** UV absorption and Fl emission of AN-2S and AN-4S probes.

Probe	Solvent (*V*(DMF):*V*(PBS))	*λ*_ex_/*λ*_em_ (nm)	Detection Mode
AN-2S	1:9	360/425	Turn-off
AN-2S + Hg^2+^	1:9	410/510	Turn-on
AN-4S	1:99	360/445	Turn-off
AN-4S + Hg^2+^	1:99	430/510	Turn-on

**Table 2:** Results of fluorescence titration of AN-2S and AN-4S probes.

Probe	Detection Mode	Linear Response Range (μmol/L)	σ	LOD (mol/L)
AN-2S	Turn-off	0~45	12.31	2.5 × 10^−7^
AN-2S	Turn-on	0~45	4.99	9.8 × 10^−8^
AN-4S	Turn-off	0~40	16.83	2.3 × 10^−7^
AN-4S	Turn-on	0~40	2.92	4.9 × 10^−8^

**Table 3:** Response of AN-4S to Hg^2+^ in simulated natural water when *λ*_ex_ = 360 nm and *λ*_ex_ = 430 nm.

Sample	pH	*λ*_ex_/*λ*_em_ (nm)	Added Hg^2+^ Concentration (μmol/L)	Detected Hg^2+^ Concentration (μmol/L)	Recovery (%)	RSD (%, *n* = 3)
Lake water	6.8	360/445	5	4.51	90.20	1.98
			10	11.49	114.90	3.45
			20	21.49	107.45	2.39
			30	30.45	101.50	1.96
		430/510	5	4.48	89.60	2.82
			10	10.33	103.30	3.74
			20	20.89	104.45	4.25
			30	28.02	101.61	1.77

**Table 4:** Comparison of probes in this work with other Hg^2+^ probes.

	Probe	Solvent	LOD (mol/L)	*c*_probe_ (μnol/L)	Liner Range (μnol/L)	Application
1 [32]	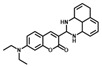	50% MeCN	1.1 × 10^−6^	10	0~0.12	Cell imaging
2 [33]	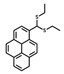	50% MeCN	5.7 × 10^−8^	10	-	Cell imaging
3 [34]	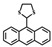	20% DMF	2.3 × 10^−8^	10	1~10	Cell imaging
4 [36]	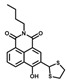	10% DMSO	1.3 × 10^−8^	10	1~10	Cell imaging
5 [37]	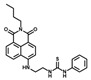	90% EtOH	3.3 × 10^−7^	10	1~10	Cell imaging
6 [38]	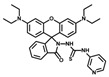	10% DMF	1.3 × 10^−7^	10	0~10	-
AN-2S	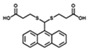	10% DMF	2.5 × 10^−7^ (*λ*_ex_ = 360 nm)/ 9.8 × 10^−8^ (*λ*_ex_ = 410 nm)	100	0~50	Test strip
AN-4S	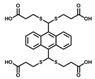	1% DMF	2.3 × 10^−7^ (*λ*_ex_ = 360 nm)/ 4.9 × 10^−8^ (*λ*_ex_ = 410 nm)	100	0~35	Test strip

## Data Availability

The data presented in this study are available in this article and the Supplementary Materials.

## Supplementary Materials

The following supporting information can be downloaded at: https://www.mdpi.com/article/10.3390/molecules30030561/s1, Figure S1: ^1^H NMR spectrum (400 MHz, DMSO-*d*_6_, 298 K) of AN-2S; Figure S2: ^13^C NMR spectrum (100 MHz, DMSO-*d*_6_, 298 K) of AN-2S; Figure S3: ^1^H NMR spectrum (400 MHz, DMSO-*d*_6_, 298 K) of recovered 9-AN; Figure S4: ^13^C NMR spectrum (100 MHz, DMSO-*d*_6_, 298 K) of recovered 9-AN; Figure S5: ^1^H NMR spectrum (400 MHz, DMSO-*d*_6_, 298 K) of AN-4S; Figure S6: ^13^C NMR spectrum (100 MHz, DMSO-*d*_6_, 298 K) of AN-4S; Figure S7: ^1^H NMR spectrum (400 MHz, DMSO-*d*_6_, 298 K) of recovered AN-DC; Figure S8: ^13^C NMR spectrum (100 MHz, DMSO-*d*_6_, 298 K) of recovered AN-DC; Figure S9: Mass spectrum of AN-2S, as measured by MALDI-TOF mass spectrometry; Figure S10: Mass spectrum of AN-4S, as measured by MALDI-TOF mass spectrometry; Figure S11: Fluorescence image of AN-2S probe (100 μmol/L) in different solvents; Figure S12: Temperature stability of (a) AN-2S (*λ*_ex_ = 360 nm, *c*(AN-2S) = 100 μmol/L, *c*(Hg^2+^) = 100 μmol/L) and (b) AN-4S (*λ*_ex_ = 360 nm, *c*(AN-4S) = 100 mol/L, *c*(Hg^2+^) = 200 μmol/L); Figure S13: Influence of anions on AN-2S response to Hg^2+^ when (a) *λ*_ex_ = 360 nm and (b) *λ*_ex_ = 410 (*c*(AN-2S) = 100 μmol/L, *c*(anion) = *c*(Hg^2+^) = 100 μmol/L); Figure S14: Influence of anions on AN-4S response to Hg^2+^ when (a) *λ*_ex_ = 360 nm and (b) *λ*_ex_ = 430 (*c*(AN-4S) = 100 μmol/L, *c*(anion) = *c*(Hg^2+^) = 200 μmol/L); Figure S15: (a) ^1^H NMR and (b) FT-IR spectra of AN-2S, AN-2S + Hg^2+^ and 9-AN; Figure S16: Response diagram of AN-2S (100 μmol/L) to Hg^2+^ in simulated natural water samples when (a) *λ*_ex_ =360 nm and (b) *λ*_ex_ =410 nm; Table S1: Response of AN-2S to Hg^2+^ in simulated natural water when *λ*_ex_ =360 nm and *λ*_ex_ =410 nm; Figure S17: Images of the test strip under sunlight and 360 nm UV light before and after immersed in water samples; Figure S18: (a) Optimized molecular configurations for AN-2S and 9-AN probes, and (b) HOMO–LUMO energy gap between AN-2S and 9-AN probes; Figure S19: Fluorescence ability of AN-2S at (a) *λ*_ex_ = 360 and (b) *λ*_ex_ = 410 after prolonged placement (*c*(AN-2S) = 100 μmol/L, *c*(Hg^2+^) = 100 μmol/L); fluorescence ability of AN-4S at (c) *λ*_ex_ = 360 and (d) *λ*_ex_ = 430 after prolonged placement (*c*(AN-4S) = 100 μmol/L, *c*(Hg^2+^) = 200 μmol/L); Figure S20: Fluorescence ability of AN-2S at (a) *λ*_ex_ = 360 and (b) *λ*_ex_ = 410 after 360 nm UV illumination (*c*(AN-2S) = 100 μmol/L, *c*(Hg^2+^) = 100 μmol/L); fluorescence ability of AN-4S at (c) *λ*_ex_ = 360 and (d) *λ*_ex_ = 430 after 360 nm UV illumination (*c*(AN-4S) = 100 μmol/L, *c*(Hg^2+^) = 200 μmol/L); Table S2: Molecular coordinates of AN-2S calculated by DFT simulations; Table S3: Molecular coordinates of 9-AN calculated by DFT simulations; Table S4: Molecular coordinates of AN-4S calculated by DFT simulations; Table S5. Molecular coordinates of AN-DC calculated by DFT simulations. All calculations were carried out with the Gaussian 09 software [39]. The B3LYP functional [40] was adopted for all calculations in combination with the D3BJ dispersion correction [41]. For geometry optimization, the 6-31G(d) basis set was used [42,43,44]. The SMD implicit solvation model6 was used to account for the solvation effect.

## Abbreviations

The following abbreviations are used in this manuscript:

9-AN	9-Anthraformaldehyde
AN-DC	9, 10-Anthradialdehyde
TFA	Trifluoroacetic acid
LOD	Limit of detection
ICT	Intramolecular charge transfer
DFT	Density functional theory
