# Supplementary material for: An Anthracene-Based Hg2+ Fluorescent Probe with Dithioacetal: Simple Synthesis, High Selectivity and Sensitivity, and Dual-Mode Detection Capability
Source: Molecules. 2025 Jan 26;30(3):561. doi: 10.3390/molecules30030561 (PMC11820999; doi:10.3390/molecules30030561)
Supplement: Supplementary file 1 [file molecules-30-00561-s001.zip › molecules-3438660-supplementary.pdf]

Supporting materials

An Anthracene-Based Hg^{2+} Fluorescent Probe with Dithioacetal: Simple Synthesis, High Selectivity, Sensitivity, and Dual-Mode Detection

Hongli Ren 1, and Qiang Yan 1, *
(State Key Laboratory of Polymer Molecular Engineering of Polymers, Department of Macromolecular Science, Fudan University, Shanghai 200438, China)

Contents

Figure S1. ^{1}H NMR spectrum (400 MHz, DMSO-d_6, 298 K) of AN-2S 3

Figure S2. ^{13}C NMR spectrum (100 MHz, DMSO-d_6, 298 K) of AN-2S 3

Figure S3. ^{1}H NMR spectrum (400 MHz, DMSO-d_6, 298 K) of recovered 9-AN.................. 4

Figure S4. ^{13}C NMR spectrum (100 MHz, DMSO-d_6, 298 K) of recovered 9-AN................. 4

Figure S5. ^{1}H NMR spectrum (400 MHz, DMSO-d_6, 298 K) of AN-4S 5

Figure S6. ^{13}C NMR spectrum (100 MHz, DMSO-d_6, 298 K) of AN-4S 5

Figure S7. ^{1}H NMR spectrum (400 MHz, DMSO-d_6, 298 K) of recovered AN-DC 6

Figure S8. ^{13}C NMR spectrum (100 MHz, DMSO-*d*6, 298 K) of recovered AN-DC 6

Figure S9. Mass spectrum of AN-2S measured by MALDI-TOF... 7

Figure S10. Mass spectrum of AN-4S measured by MALDI-TOF.. 7

Figure S11. Fluorescence image of probe AN-2S (100 μmol/L) in different solvents............. 8

Figure S12. Temperature stability of (a) AN-2S (λ_{ex}=360 nm, c(AN-2S)=100 μmol/L, c(Hg^{2+})=100 μmol/L) and (b) AN-4S (λ_{ex}=360 nm, c(AN-4S)=100 mol/L, c(Hg^{2+})=200 μmol/L)... 8

Figure S13. Influence of anions on AN-2S response to Hg^{2+} when (a) λ_{ex}=360 nm and (b) λ_{ex}=410 (c(AN-2S)=100 μmol/L, c(anion)=c(Hg^{2+})=100 μmol/L) 9

Figure S14. Influence of anions on AN-4S response to Hg^{2+} when (a) λ_{ex}=360 nm and (b) λ_{ex}=430 (c(AN-4S)=100 μmol/L, c(anion)=c(Hg^{2+})=200 μmol/L) 9

Figure S15. (a) ^{1}H NMR and (b) FT-IR spectra of AN-2S, AN-2S+Hg^{2+}, and 9-AN............ 10

Figure S16. Response diagram of AN-2S (100 μmol/L) to Hg^{2+} in simulated natural water samples when (a) λ_{ex}=360 nm and (b) λ_{ex}=410 nm.. 11

Table S1. Response of AN-2S to Hg^{2+} in simulated natural water when λ_{ex}=360 nm and λ_{ex}=410 nm 11

Figure S17. Images of the test strip under sonlight and 360 nm UV light before and after immersed in water samples 11

Figure S18. (a) Molecular configurations optimized for probes AN-2S and 9-AN, (b) HOMO-LUMO orbital energy gap between probes AN-2S and 9-AN........ 12

Figure S19. Fluorescence ability of AN-2S at (a) λ_{ex}=360 and (b) λ_{ex}=410 after prolonged placement (c(AN-2S)=100 μmol/L, $c(Hg^{2+})$=100 μmol/L); fluorescence ability of AN-4S at (c) λ_{ex}=360 and (d) λ_{ex}=430 after prolonged placement (c(AN-4S)=100 μmol/L, $c(Hg^{2+})$=200 μmol/L)........ 13

Figure S20. Fluorescence ability of AN-2S at (a) λ_{ex}=360 and (b) λ_{ex}=410 after 360 nm UV illumination (c(AN-2S)=100 μmol/L, $c(Hg^{2+})$=100 μmol/L); fluorescence ability of AN-4S at (c) λ_{ex}=360 and (d) λ_{ex}=430 after 360 nm UV illumination (c(AN-4S)=100 μmol/L, $c(Hg^{2+})$=200 μmol/L) 13

Table S2. Molecular coordinates of AN-2S calculated by DFT........ 14

Table S3. Molecular coordinates of 9-AN calculated by DFT 16

Table S4. Molecular coordinates of AN-4S calculated by DFT........ 17

Table S5. Molecular coordinates of AN-DC calculated by DFT........ 21

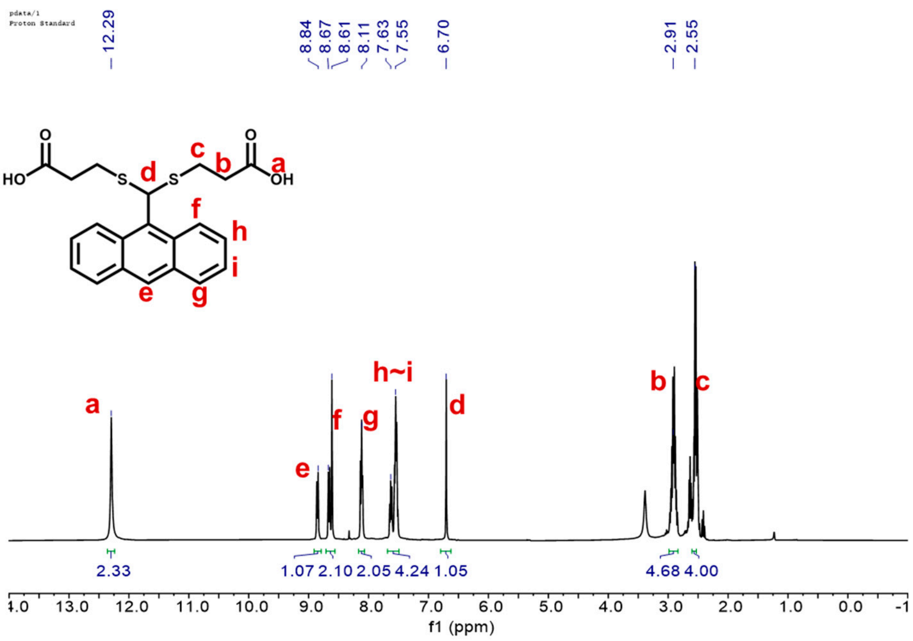

Figure S1. ^{1}H NMR spectrum (400 MHz, DMSO-d_6, 298 K) of AN-2S

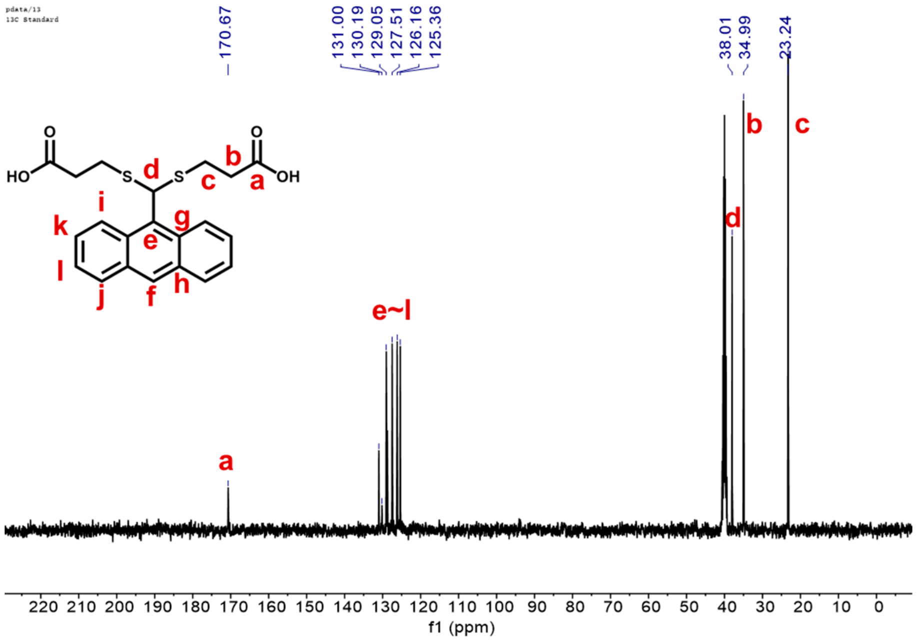

Figure S2. ^{13}C NMR spectrum (100 MHz, DMSO-d_6, 298 K) of AN-2S

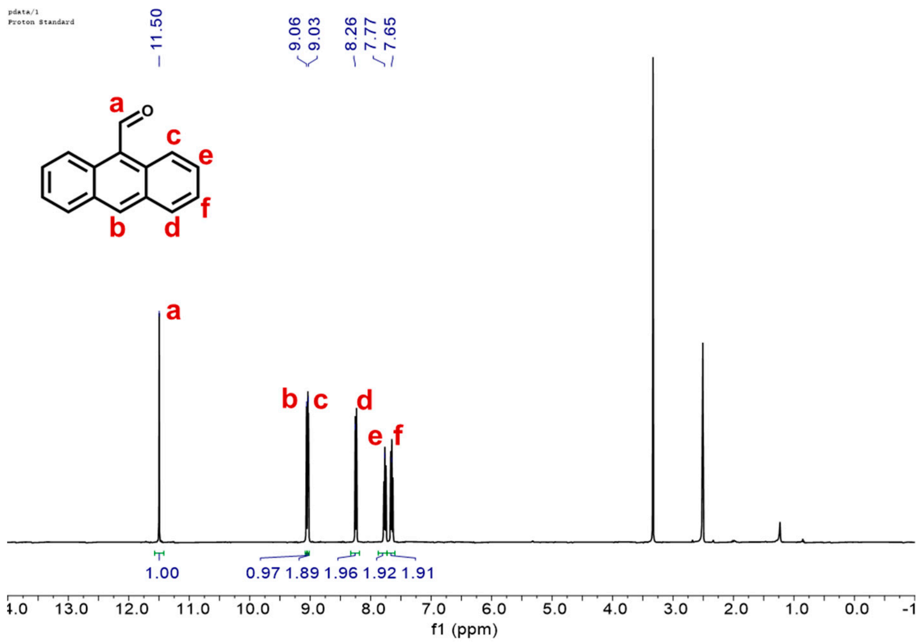

Figure S3. ^{1}H NMR spectrum (400 MHz, DMSO-d_6, 298 K) of recovered 9-AN

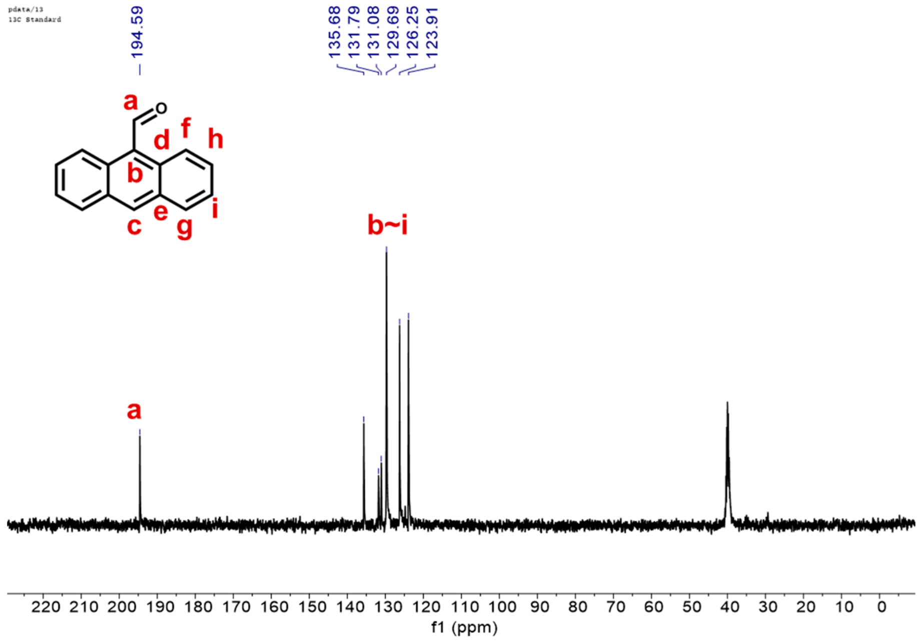

Figure S4. ^{13}C NMR spectrum (100 MHz, DMSO-d_6, 298 K) of recovered 9-AN

^{1}H NMR (400 MHz, DMSO-d_6, 298 K): δ = 11.50 (s, 1H), 9.06 (d, J = 7.9, 2H), 9.03 (s, 1H), 8.26 (d, J = 7.6, 2H), 7.77 (m, 2H), 7.65 (m, 2H). ^{13}C NMR (100 MHz, DMSO-d_6, 298 K): δ = 194.6, 135.7, 131.8, 131.1, 129.7, 126.3, 123.9.

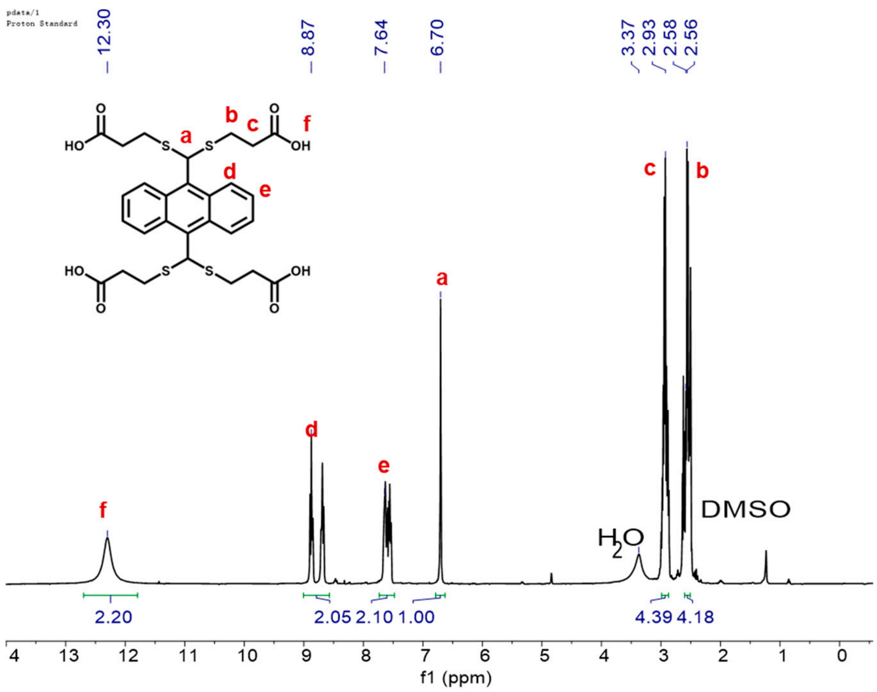

Figure S5. ^{1}H NMR spectrum (400 MHz, DMSO-d_6, 298 K) of AN-4S

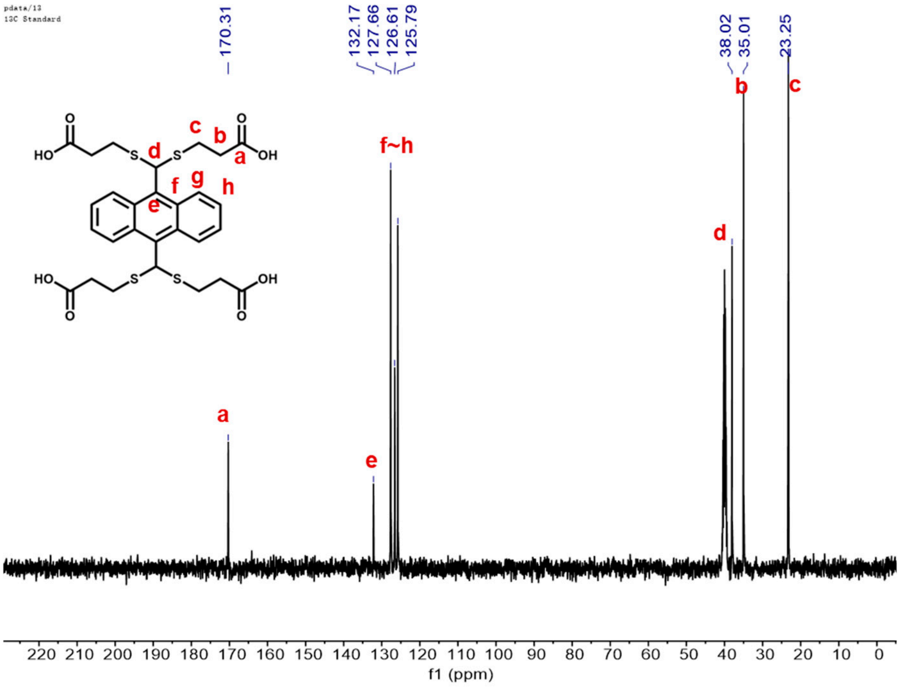

Figure S6. ^{13}C NMR spectrum (100 MHz, DMSO-d_6, 298 K) of AN-4S

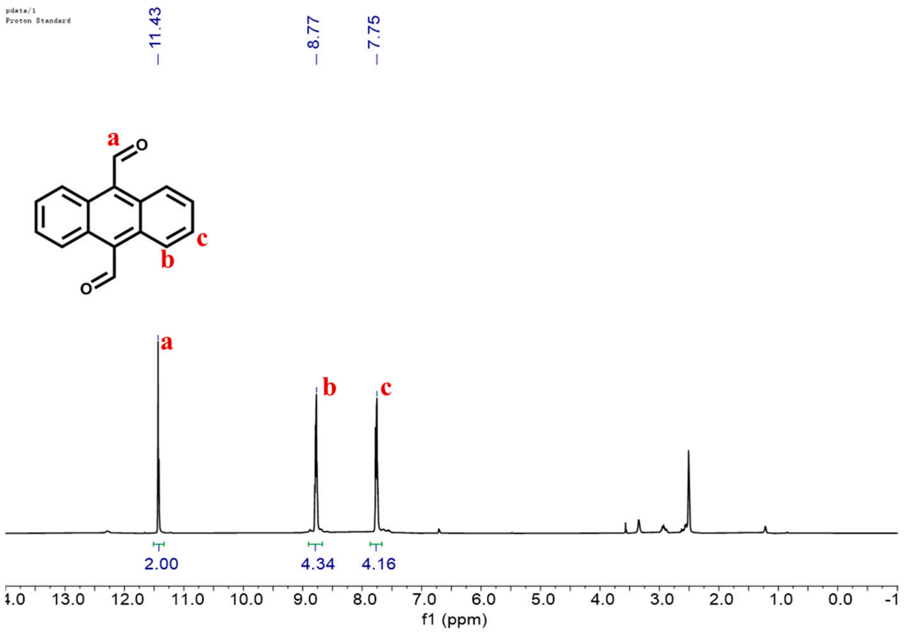

Figure S7. ^{1}H NMR spectrum (400 MHz, DMSO-d_6, 298 K) of recovered AN-DC

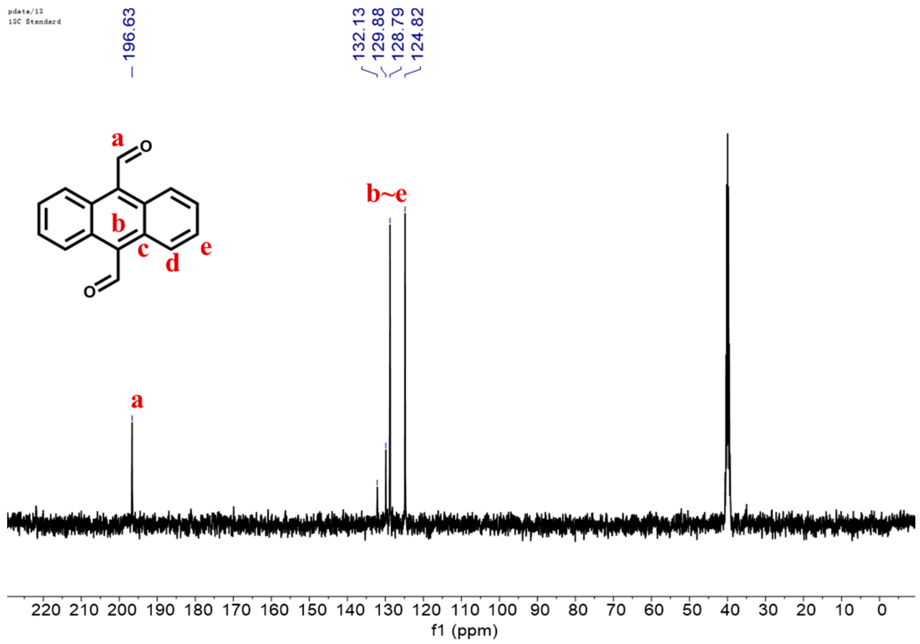

Figure S8. ^{13}C NMR spectrum (100 MHz, DMSO-*d*6, 298 K) of recovered AN-DC

^{1}H NMR (400 MHz, DMSO-d_6, 298 K): δ = 11.43 (s, 2H), 8.77 (m, 4H), 7.75 (m, 4H). ^{13}C NMR (100 MHz, DMSO-d_6, 298 K): 196.6, 132.1, 129.9, 128.8, 124.8.

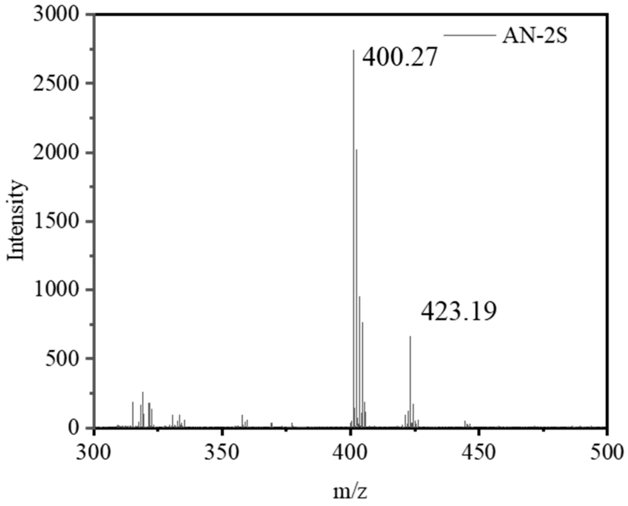

Figure S9. Mass spectrum of AN-2S measured by MALDI-TOF

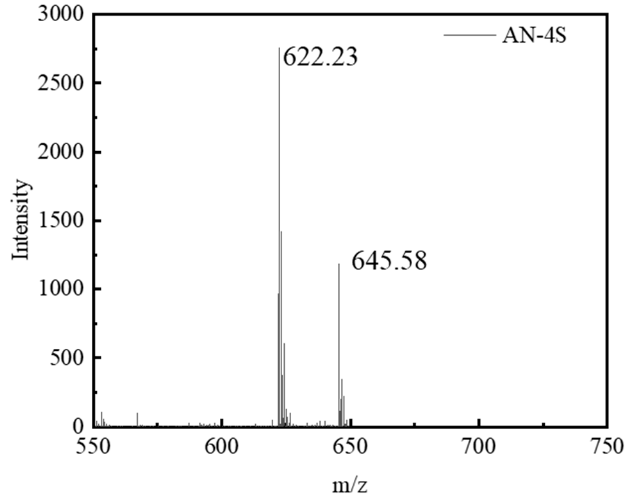

Figure S10. Mass spectrum of AN-4S measured by MALDI-TOF

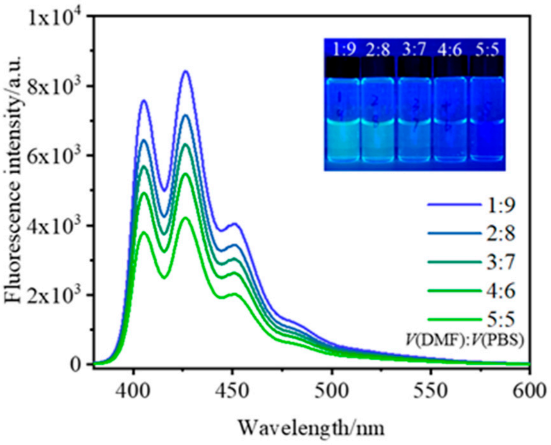

Figure S11. Fluorescence spectrum of probe AN-2S (100 μmol/L λ_{ex}=360 nm) in different solvents

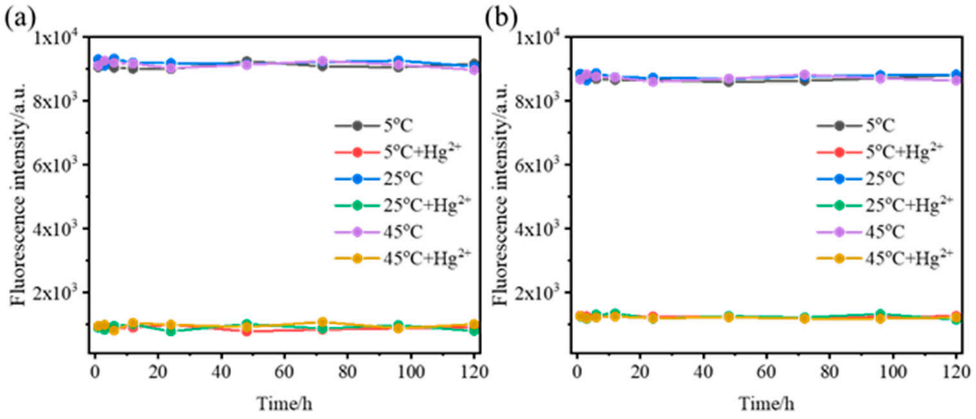

Figure S12. Temperature stability of (a) AN-2S (λ_{ex}=360 nm, c(AN-2S)=100 μmol/L, $c(Hg^{2+})$=100 μmol/L) and (b) AN-4S (λ_{ex}=360 nm, c(AN-4S)=100 mol/L, $c(Hg^{2+})$=200 μmol/L)

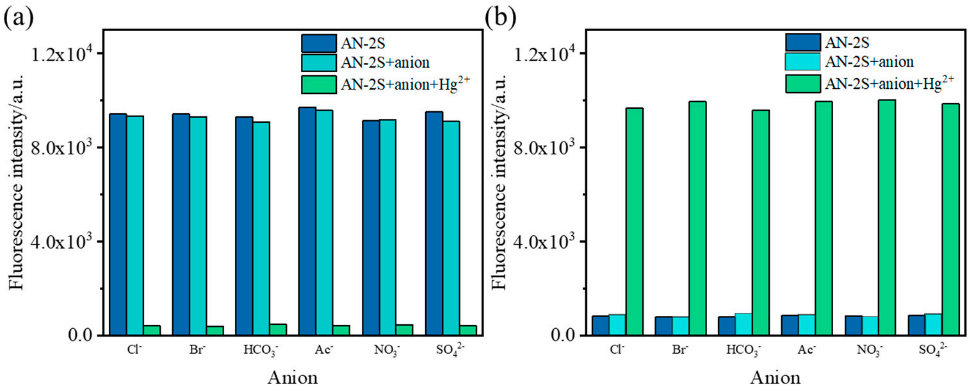

Figure S13. Influence of anions on AN-2S response to Hg^{2+} when (a) λ_{ex}=360 nm and (b) λ_{ex}=410 (c(AN-2S)=100 μmol/L, c(anion)=c(Hg^{2+})=100 μmol/L)

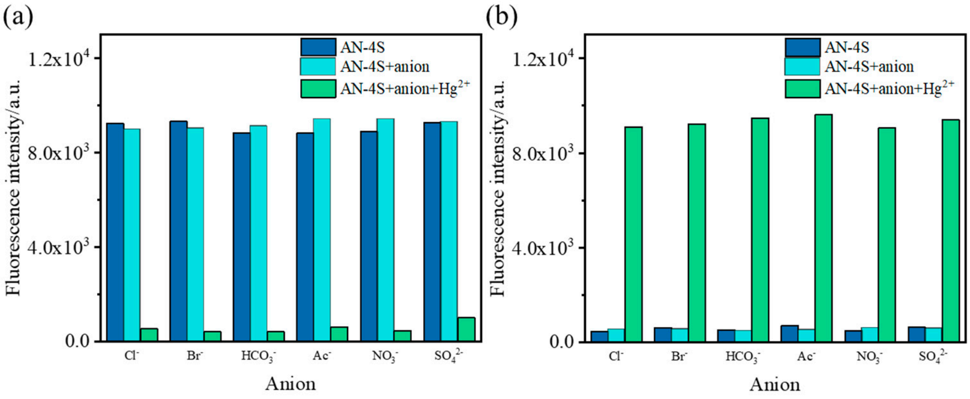

Figure S14. Influence of anions on AN-4S response to Hg^{2+} when (a) λ_{ex}=360 nm and (b) λ_{ex}=430 (c(AN-4S)=100 μmol/L, c(anion)=c(Hg^{2+})=200 μmol/L)

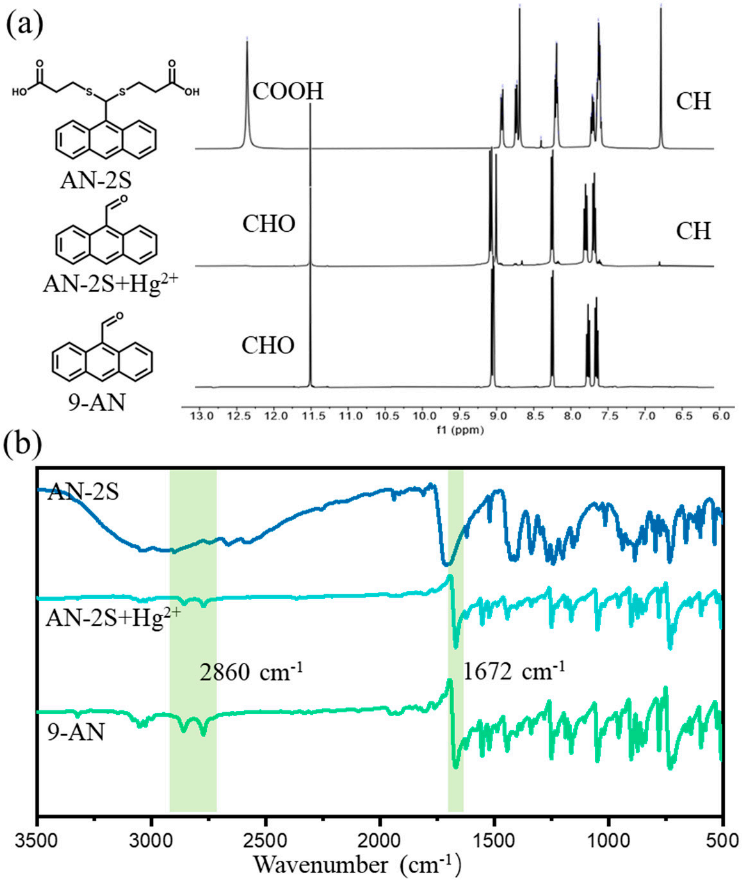

Figure S15. (a) ^{1}H NMR and (b) FT-IR spectra of AN-2S, AN-2S+Hg^{2+}, and 9-AN

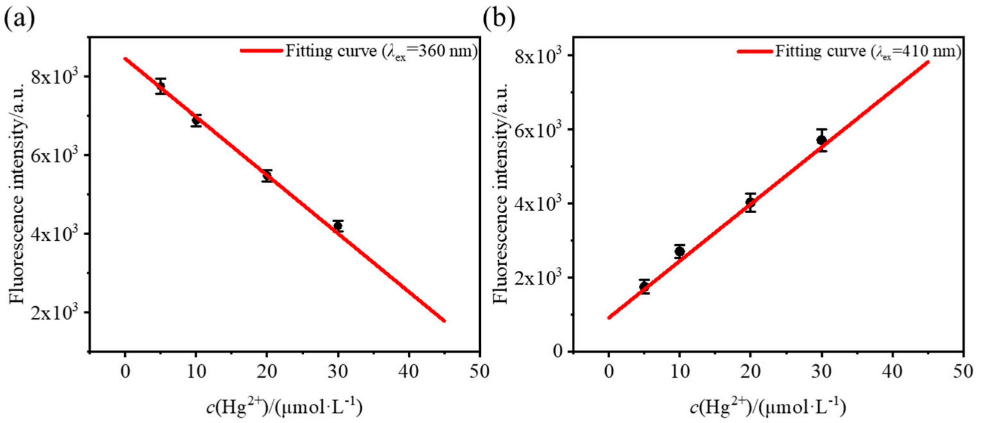

Figure S16. Response diagram of AN-2S (100 μmol/L) to Hg^{2+} in simulated natural water samples when (a) λ_{ex}=360 nm and (b) λ_{ex}=410 nm

Table S1. Response of AN-2S to Hg^{2+} in simulated natural water when λ_{ex}=360 nm and λ_{ex}=410 nm

Sample	pH	Ex/Em (nm)	Added Hg^{2+} (10^{-6} M)	Found Hg^{2+} (10^{-6} M)	Recovery (%)	RSD (%)
River water	6.8	360/425	5	4.75	95.00	2.44
			10	10.63	106.30	2.09
			20	20.13	100.65	2.71
			30	28.77	95.90	3.14
		410/510	5	5.45	109.00	2.49
			10	11.67	116.70	6.52
			20	20.37	101.85	6.13
			30	31.37	100.36	5.34

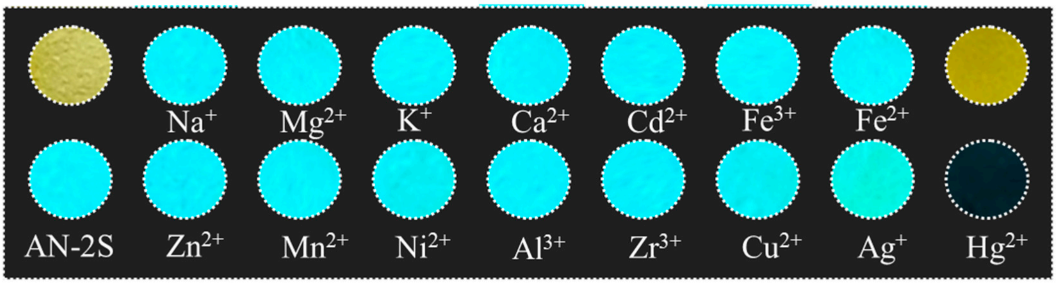

Figure S17. Images of the test strip under sonlight and 360 nm UV light before and after immersed in water samples

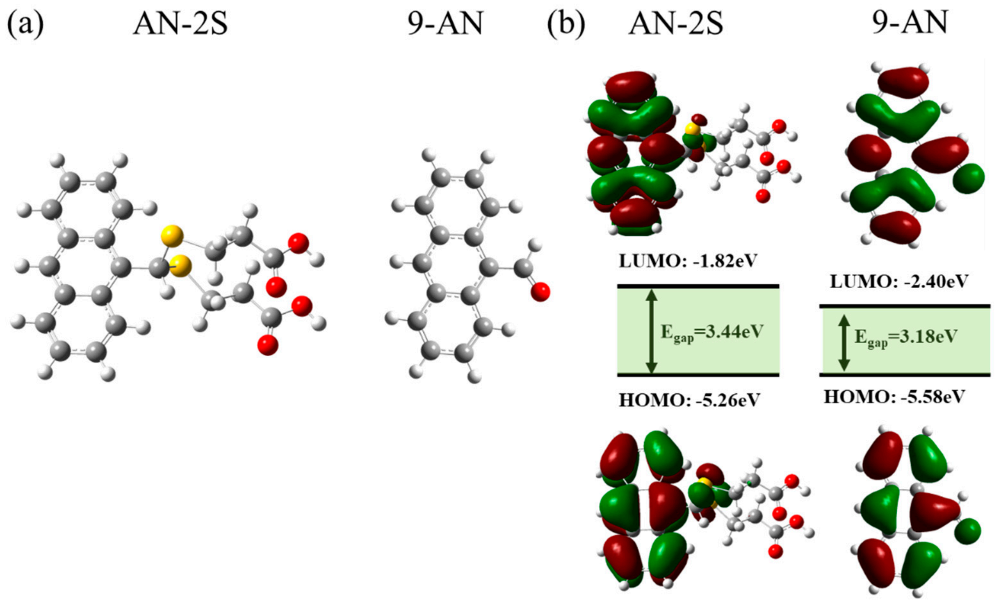

Figure S18. (a) Molecular configurations optimized for probes AN-2S and 9-AN, (b) HOMO-LUMO orbital energy gap between probes AN-2S and 9-AN

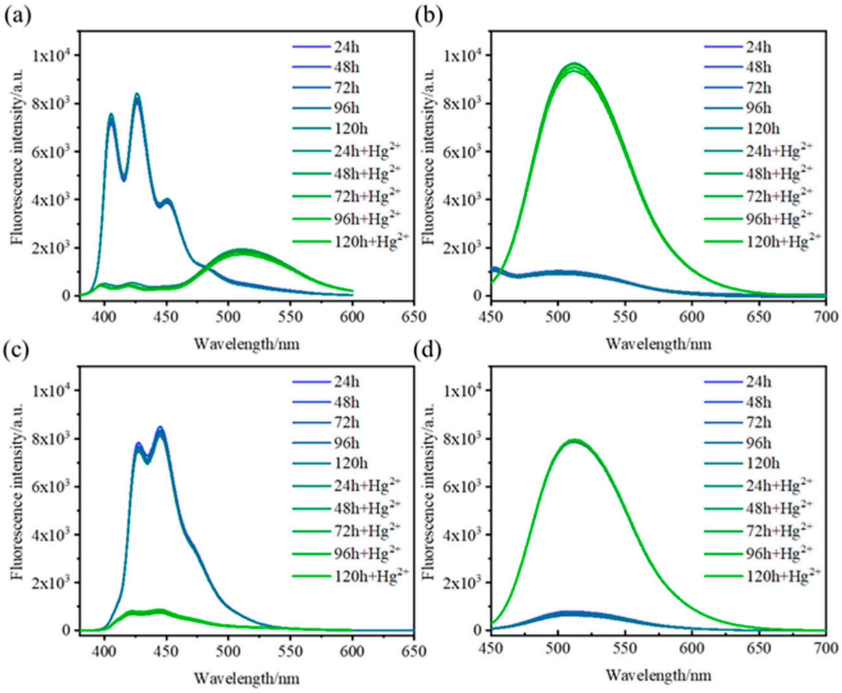

Figure S19. Fluorescence ability of AN-2S at (a) λ_{ex}=360 and (b) λ_{ex}=410 after prolonged placement (c(AN-2S)=100 μmol/L, $c(Hg^{2+})$=100 μmol/L); fluorescence ability of AN-4S at (c) λ_{ex}=360 and (d) λ_{ex}=430 after prolonged placement (c(AN-4S)=100 μmol/L, $c(Hg^{2+})$=200 μmol/L)

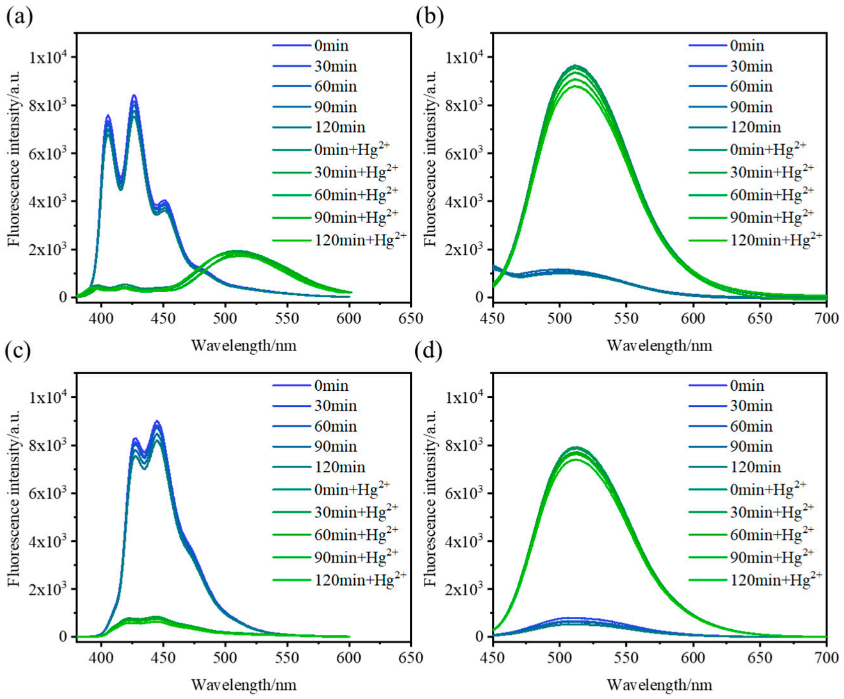

Figure S20. Fluorescence ability of AN-2S at (a) λ_{ex}=360 and (b) λ_{ex}=410 after 360 nm UV illumination (c(AN-2S)=100 μmol/L, $c(Hg^{2+})$=100 μmol/L); fluorescence ability of AN-4S at (c) λ_{ex}=360 and (d) λ_{ex}=430 after 360 nm UV illumination (c(AN-4S)=100 μmol/L, $c(Hg^{2+})$=200 μmol/L)

DFT calculation method

All calculations were carried out with the Gaussian 09 software [39]. The B3LYP functional [40] was adopted for all calculations in combination with the D3BJ dispersion correction [41]. For geometry optimization, the 6-31G(d) basis set was used [42–44]. The SMD implicit solvation model6 was used to account for the solvation effect.

Table S2. Molecular coordinates of AN-2S calculated by DFT

		x	y	z
1	C	3.905000	3.781000	0.366999
2	C	2.504000	3.734999	0.608999
3	C	1.811000	2.555000	0.531000
4	C	2.462000	1.320999	0.202999
5	C	3.889000	1.378999	-0.040000
6	C	4.574000	2.629999	0.051000
7	C	1.794999	0.073000	0.107000
8	C	2.520000	-1.101000	-0.216999
9	C	3.942000	-1.020000	-0.458999
10	C	4.586999	0.214000	-0.363000
11	C	1.909000	-2.393000	-0.333000
12	C	2.641000	-3.504000	-0.661999
13	C	4.042000	-3.413999	-0.897000
14	C	4.670000	-2.203000	-0.795000
15	C	0.305000	0.001999	0.351999
16	S	-0.616000	-0.562000	-1.171000
17	S	-0.008000	-0.931999	1.925000
18	C	-1.728999	-0.419999	2.298000
19	C	-1.962000	0.695000	-1.222000
20	C	-2.789000	-1.299000	1.630999
21	C	-3.221999	0.092000	-1.838000
22	C	-4.405000	1.014999	-1.685999
23	O	-5.575000	0.369000	-1.877999
24	C	-4.100999	-0.586000	1.420000
25	O	-5.054000	-1.423000	0.956999
26	O	-4.301999	0.606999	1.579999
27	O	-4.345000	2.205000	-1.427000

28	H	4.434000	4.727000	0.433999
29	H	4.974289	4.070959	0.260641
30	H	1.975000	4.649999	0.860000
31	H	3.029458	2.793430	0.333120
32	H	0.745999	2.576999	0.726000
33	H	2.629347	1.854990	0.249819
34	H	1.416570	0.956340	0.316437
35	H	4.992204	1.231778	-0.045861
36	H	5.644000	2.640000	-0.137999
37	H	3.975031	3.536291	0.293160
38	H	2.494035	-0.767213	-0.103143
39	H	1.546883	-0.753169	0.196313
40	H	5.049991	-1.045104	-0.561436
41	H	5.657000	0.269999	-0.547000
42	H	3.524928	0.122161	-0.043094
43	H	0.848000	-2.504000	-0.144000
44	H	2.688738	-1.901875	-0.957158
45	H	2.145999	-4.468000	-0.741999
46	H	3.057234	-2.476891	-0.764790
47	H	4.603000	-4.307000	-1.155000
48	H	5.122757	-3.352389	-1.155721
49	H	5.739000	-2.112000	-0.970000
50	H	4.120000	-3.170474	-0.778804
51	H	-0.094999	0.993000	0.535000
52	H	-1.823999	-0.460999	3.385999
53	H	-1.841000	0.626000	2.005000
54	H	-2.169000	1.023000	-0.203999
55	H	-1.628000	1.563000	-1.793999
56	H	-2.455999	-1.621999	0.637999
57	H	-2.967999	-2.214999	2.203000
58	H	-3.088999	-0.102000	-2.910000
59	H	-3.476000	-0.869000	-1.381000
60	H	-4.107154	2.051107	-1.409344
61	H	-6.295999	1.024999	-1.789000

62	H	-4.096784	0.358025	2.009548
63	H	-5.847000	-0.887000	0.753000

Table S3. Molecular coordinates of 9-AN calculated by DFT

		x	y	z
1	C	2.307290	-1.408564	-2.335998
2	C	1.484627	-2.018831	-1.208909
3	C	0.090314	-1.406116	-1.208909
4	C	0.203248	0.112691	-1.208909
5	C	1.396835	0.532977	-2.056399
6	C	2.634585	-0.230497	-1.604058
7	C	-0.663242	-1.859121	0.034662
8	C	-1.586645	-0.742449	0.503596
9	C	-1.039213	0.599326	0.035101
10	C	0.394920	0.607476	0.218669
11	C	-1.666709	-0.754965	2.024438
12	C	-2.471122	0.447518	2.500342
13	C	-1.445801	1.438295	2.340776
14	C	-1.662873	1.717808	0.859465
15	C	1.063078	1.964256	0.398231
16	O	1.158820	2.261555	1.764998
17	H	3.199358	-2.017596	-2.604442
18	H	1.824420	-1.289374	-3.331688
19	H	1.406146	-3.118608	-1.360893
20	H	1.980501	-1.812954	-0.233977
21	H	-0.460447	-1.737210	-2.117649
22	H	-0.726504	0.553871	-1.632808
23	H	1.190737	0.304415	-3.126003
24	H	1.570530	1.625814	-1.936794
25	H	3.585237	0.241515	-1.939071
26	H	2.819708	-0.327547	-0.510861
27	H	-1.266946	-2.762562	-0.206390
28	H	0.064047	-2.102612	0.841218

29	H	-2.602951	-0.897797	0.077265
30	H	-1.284692	0.754545	-1.039336
31	H	0.979614	0.124978	1.033591
32	H	-2.165173	-1.691242	2.361616
33	H	-0.639657	-0.706638	2.450580
34	H	-2.798425	0.344783	3.559156
35	H	-3.429072	0.640646	1.967612
36	H	-1.625429	2.337698	2.971312
37	H	-0.417345	1.134335	2.638524
38	H	-2.754404	1.769553	0.648153
39	H	-1.186098	2.686992	0.590882
40	H	2.082664	1.936959	-0.047265
41	H	0.456070	2.746680	-0.109828

Table S4. Molecular coordinates of AN-4S calculated by DFT

		x	y	z
1	C	0.766000	2.306999	3.261000
2	C	-0.644000	2.158999	3.268000
3	C	-1.262000	1.389999	2.318999
4	C	-0.525000	0.707000	1.295999
5	C	0.916999	0.845000	1.300000
6	C	1.513999	1.671000	2.307999
7	C	-1.145999	-0.090000	0.305000
8	C	-0.359000	-0.767000	-0.653000
9	C	1.082999	-0.628000	-0.649999
10	C	1.704000	0.171000	0.339999
11	C	-0.956000	-1.601000	-1.655000
12	C	-0.208000	-2.246000	-2.601999
13	C	1.201999	-2.098000	-2.611000

14	C	1.819999	-1.318000	-1.670000
15	C	3.209999	0.291000	0.365999
16	S	3.740000	1.994000	-0.113000
17	S	3.987000	-0.346000	1.940999
18	C	4.980000	-1.708000	1.223000
19	C	5.489999	1.623000	-0.500999
20	C	4.130000	-2.934000	0.846000
21	C	6.188999	2.895000	-0.964000
22	C	4.966000	-3.938999	0.096999
23	C	7.639000	2.643999	-1.291000
24	O	5.189999	-3.550000	-1.180000
25	O	8.250999	3.762999	-1.735000
26	O	5.424000	-4.970000	0.558000
27	O	8.217000	1.576999	-1.176999
28	C	-2.654000	-0.211000	0.276000
29	S	-3.366000	0.447999	-1.319000
30	S	-3.143999	-1.927999	0.779000
31	C	-4.882999	-1.654000	1.293999
32	C	-4.750000	1.468000	-0.658000
33	C	-5.885000	-1.748000	0.142000
34	C	-5.872000	1.548000	-1.687999
35	C	-7.149000	2.084000	-1.089000
36	O	-8.114999	2.225999	-2.021000
37	C	-7.193000	-1.068999	0.459999
38	O	-8.016000	-1.038999	-0.610999
39	O	-7.499000	-0.579999	1.534000
40	O	-7.328000	2.343000	0.089000

41	H	1.252000	2.925999	4.009000
42	H	0.861564	1.945846	4.309430
43	H	-1.237000	2.657999	4.029000
44	H	0.105303	1.630537	2.637094
45	H	-2.340000	1.302000	2.365000
46	H	-0.370860	1.267676	1.663503
47	H	-1.550236	0.504705	0.912935
48	H	1.715888	0.705302	0.537746
49	H	2.586000	1.817000	2.311000
50	H	0.670614	0.954698	2.427923
51	H	-0.988662	-0.977947	-0.347353
52	H	-1.187493	-0.523397	0.049158
53	H	2.107995	-0.335168	-0.329996
54	H	1.585606	1.226143	0.673802
55	H	-2.029000	-1.742999	-1.658999
56	H	-0.063910	-0.942343	-1.750476
57	H	-0.694000	-2.871999	-3.344999
58	H	-0.493098	-1.963585	-3.640137
59	H	1.795999	-2.603999	-3.366000
60	H	0.234090	-1.865920	-2.112944
61	H	2.897000	-1.233000	-1.719000
62	H	0.937285	-1.030712	-1.055956
63	H	3.645000	-0.349000	-0.390999
64	H	5.715999	-1.977000	1.985999
65	H	5.523000	-1.324999	0.356000
66	H	5.970000	1.227999	0.398000
67	H	5.527999	0.857000	-1.280999

68	H	3.297000	-2.633000	0.204999
69	H	3.732000	-3.409000	1.745000
70	H	5.711000	3.315000	-1.857000
71	H	6.149999	3.675000	-0.195000
72	H	5.083490	-4.876696	0.684953
73	H	7.944008	1.585927	-1.129066
74	H	5.769999	-4.215000	-1.601000
75	H	9.184000	3.539000	-1.925000
76	H	-3.097000	0.431999	1.026999
77	H	-5.094000	-2.412999	2.050999
78	H	-4.938000	-0.682000	1.792000
79	H	-5.118000	1.008000	0.258999
80	H	-4.381999	2.465000	-0.405999
81	H	-5.487999	-1.293999	-0.770999
82	H	-6.093999	-2.793999	-0.110999
83	H	-5.596999	2.172999	-2.545000
84	H	-6.108999	0.555999	-2.091999
85	H	-7.008555	2.410851	-0.034385
86	H	-8.920000	2.547999	-1.570000
87	H	-7.258462	-0.829766	1.545011
88	H	-8.828999	-0.562000	-0.349000

Table S5. Molecular coordinates of AN-DC calculated by DFT

		x	y	z
1	C	3.591000	-0.707000	0.000999
2	C	2.407000	-1.397000	0.000000
3	C	1.145000	-0.724000	0.000000
4	C	1.145000	0.724000	0.000000
5	C	2.406000	1.397000	0.000000
6	C	3.591000	0.707000	0.000999
7	C	-0.091000	-1.431999	0.000000
8	C	-1.331000	-0.726000	0.000000
9	C	-1.331000	0.726000	0.000000
10	C	-0.091000	1.431999	0.000000
11	C	-2.601000	-1.394000	0.000000
12	C	-3.783000	-0.708000	0.000999
13	C	-3.784000	0.707000	0.000999
14	C	-2.601000	1.394000	0.000000
15	C	-0.144000	2.914999	-0.000999
16	O	0.803000	3.699000	-0.000999
17	C	-0.144000	-2.914999	-0.000999
18	O	0.804000	-3.698000	-0.000999
19	H	4.530999	-1.250000	0.000999
20	H	3.087176	0.266796	0.192445
21	H	2.414999	-2.474000	0.000000
22	H	2.840239	-0.372187	-0.028839
23	H	0.686522	-1.738183	0.000000
24	H	0.687142	1.738463	0.000000
25	H	2.414999	2.474000	0.000000
26	H	2.795316	0.354721	-0.029290
27	H	4.530999	1.251000	0.000999
28	H	3.249892	-0.335952	0.187190
29	H	0.999445	-1.209949	0.019841
30	H	-0.914526	-1.758143	0.000000
31	H	-0.914526	1.758143	0.000000
32	H	0.999445	1.209949	0.019841
33	H	-2.658999	-2.472000	0.000999
34	H	-1.784740	-0.638442	-0.040278

35	H	-4.721999	-1.252000	0.000999
36	H	-3.435598	0.323607	0.233097
37	H	-4.723000	1.251000	0.000999
38	H	-3.842105	-0.380770	0.229365
39	H	-2.658999	2.470999	0.000999
40	H	-1.782862	0.640487	-0.040474
41	H	-1.147999	3.353000	-0.001999
42	H	0.907990	2.551570	0.001280
43	H	-1.146999	-3.353000	-0.001999
44	H	0.908714	-2.553672	0.001307

References:

[39] Moha R., Gossen V., USRCAT: real-time ultrafast shape recognition with pharmacophoric constraints. *J Cheminform*, **2012**, 4, 27

[40] Becke A. D., Density-functional thermochemistry. IV. A new dynamical correlation functional and implications for exact-exchange mixing. *J. Chem. Phys*, **1996**, 104, 1040—1046

[41] Grimme S., Antony J., Ehrlich S., Krieg H., A consistent and accurate ab initio parametrization of density functional dispersion correction (DFT-D) for the 94 elements H-Pu. *J. Chem. Phys.*, **2010**, 132, 154104

[42] Henriksson A., Sundbom M., Dynamic polarizabilities of polyaromatic hydrocarbons using coupled-cluster linear response theory. *Theor. Chim. Acta*, **1973**, 28, 213—222

[43] Giuliano, Alagona, Renzo, Cimiraglia, Eolo, ScroccoJacopo, Tomasi, Self—Consistent Molecular Orbital Methods. XII. Further Extensions of Gaussian—Type Basis Sets for Use in Molecular Orbital Studies of Organic Molecules. *J. Chem. Phys.,* **1972**, 56, 2257—2261

[44] Marenich A. V., Cramer C. J., Truhlar D. G., Universal Solvation Model Based on Solute Electron Density and on a Continuum Model of the Solvent Defined by the Bulk Dielectric Constant and Atomic Surface Tensions. *J. Phys. Chem. B*, **2009**, 113(18), 6378
